# The Tubercular Origin of Joint Diseases

**Published:** 1884-12

**Authors:** 


### The Tubercular Origin of Joint Diseases.

At a recent meeting of the Buffalo Medical and Surgical Association, Dr. E. R. Barnes, of this city, made the following pertinent remarks:

Mr. President—In advocating the theory of the tubercular origin of joint diseases, Dr. Park, of this city, stated, at the October meeting of this society, that Dr. Sayre knew nothing of the pathology of joint diseases, but had become prominent in this connection through the advocacy of two principles, or modes of treatment, namely, extension and fixation. In relation to Dr. Barwell's non-acceptance of the tubercular theory, he stated that both English and American surgeons had, with singular obstinacy, refused to accept as final the conclusions of continental observers.

Now, Mr. President, I am not prepared to discuss this subject in a very learned or elaborate manner, but it seems to me proper that a word should be spoken about it, as it appears to the ordinary practitioner. Dr. Sayre teaches that some injury to the joint structures, perhaps but slight and not readily traced, is, in nearly all cases, the beginning of the series of phenomena which constitute the diseases under consideration. He does not believe that in most cases the disease is of constitutional origin. If it be said that Dr. Sayre's views, as to the pathology of these diseases, are incorrect, and that he has been rendered noteworthy only by the advocacy of certain modes of treatment, I would ask, Mr. President, what has been the consequence, to the profession in this country, of such advocacy? I think it has been that we have had placed in our hands by far the most valuable means for the palliation, and for the cure, of these diseases that we have ever possessed. It is a clinical fact that in the earlier stages, before destructive changes have taken place, the mechanical application of the two principles of extension and fixation will, alone, in a majority of cases, cause the pain to cease, the inflammation to subside, and place the patient in the way to ultimate recovery. He may recover with slight local impairment and with no subsequent appearance of constitutional contamination. Now. Mr. President, I am unable to reconcile the fact of such recovery, by the aid only of mechanical means, with the theory that the disease was of constitutional origin.

I do not know how true the statement may be that English and American surgeons often unreasonably refuse to accept the conclusions of continental teachers. But I recall the fact that it was reserved for a distinguished American surgeon, Prof. Willard Parker, of New York, to establish, by his advocacy and by his practical demonstrations, though he did not originate, the method of relieving perityphlitic abscess by incision—a most valuable contribution to the resources of our art, though the learning of continental Europe had, as yet, led only to palliative measures. In this case, as in the before-mentioned advocacy by Dr. Sayre, I find it difficult to reconcile the ability to perceive and to teach the best method to treat a disease, with ignorance of the true nature of that disease.

